# From diagnosis to daily realities: longitudinal phenomenological exploration of symptom trajectories in glioma survivorship

**DOI:** 10.1007/s00520-026-11200-0

**Published:** 2026-09-21

**Authors:** Xiao-jing Meng, Zi-chen Zhang, Li Xiang, Gao Min, Li-ming Li

**Affiliations:** Henan Provincial People’s Hospital, Henan Province, No. 7 Weilu Road, Zhengzhou City, 450003 China

**Keywords:** Glioma, Living, Glioma, Needs, Symptom management

## Abstract

**Background:**

With the continuous improvement of global cancer survival rates, the long-term quality of life among cancer survivors has attracted increasing attention. Glioma is a common primary malignant tumor of the central nervous system. Both the disease itself and anti-tumor therapies induce a variety of dynamically fluctuating symptoms throughout the disease course, which impose persistent physical and psychological impairments and substantially compromise patients’ quality of life. Current qualitative studies have only preliminarily explored symptom-related issues in patients with glioma, lacking longitudinal analyses regarding the dynamic evolution of patients’ symptom perceptions, coping strategies, and care demands. Accordingly, this study adopts a longitudinal phenomenological approach to address the gaps in existing evidence.

**Aims:**

This longitudinal qualitative study aims to conduct a phenomenological exploration of the symptom trajectories in glioma survivorship, specifically focusing on the evolving processes of symptom recognition, the diversity of long-term coping strategies, the clinical challenges in symptom modulation, and the resultant needs for a structured intervention framework.

**Methods:**

Based on the phenomenological approach, semi-structured interviews were implemented among 12 glioma survivors at three time points (T1, initial diagnosis; T2, 1 month after surgery; T3, 3 months after surgery). Colaizzi’s method was used to analyze interview transcripts and extract the essential themes of patients’ long-term survivorship experience.

**Results:**

The following four themes and associated categories were identified: (1) self-perception and interpretation of the disease, (2) differentiated coping with symptoms, (3) practical dilemmas in symptom management, (4) core needs in symptom management.

**Conclusion:**

The symptom journey for glioma survivors is not static but a complex trajectory marked by significant and evolving burdens. Our findings, extending from our earlier research, underscore the critical need for healthcare systems to move beyond episodic care. Future efforts must focus on developing longitudinal, patient-centered intervention frameworks that address the unmet needs across the survivorship continuum, thereby effectively reducing symptom burden and enhancing quality of life.

## Background

The rising global cancer survival rates have shifted the focus of oncology research and practice towards understanding and improving the long-term well-being of survivors. Within this broader context, the symptom experience and management needs of the survivorship population have emerged as a critical twenty-first-century theme [1].

Gliomas, originating from neuroepithelial cells, are the most common primary malignant tumors of the central nervous system in clinical incidence, accounting for 50% to 60% of all intracranial tumors [2–4]. The fifth edition of the World Health Organization (WHO) classification of central nervous system tumors published in 2021 categorizes gliomas into Grade 1 to Grade 4. Grades 1 and 2 correspond to low-grade gliomas, while Grades 3 and 4 are high-grade gliomas. A higher grade indicates a higher degree of malignancy of the glioma. Since this grading system is primarily determined based on the morphological features of tumors, notable individual differences in prognosis still exist among patients with identical tumor grades.

Currently, clinical practice has abandoned a single treatment model and can develop individualized and differentiated treatment plans based on the severity of the patient's condition and physical status. However, even with standardized systematic treatments such as surgery, concurrent chemoradiotherapy, and adjuvant chemotherapy, the median overall survival of patients with WHO grade IV malignant glioma is still less than 2 years, and the 5-year survival rate is only about 10% [5]. Therefore, it is of great practical significance and urgent necessity to conduct research focusing on symptoms among glioma survivors [6, 7]. In addition, the disease itself and anti-tumor treatment impose a severe and dynamically changing symptom burden on patients, which substantially impairs their health-related quality of life [8].

The symptom landscape in glioma is notably complex and fluctuates significantly across the disease trajectory, from diagnosis through treatment and into long-term follow-up or end-of-life care. Patients frequently contend with a multitude of symptoms, including fatigue, drowsiness, cognitive deficits, and sleep disturbances, the prevalence and severity of which evolve over time [9, 10]. This dynamic nature of symptoms underscores a significant clinical challenge: the static, cross-sectional snapshots provided by most existing studies are insufficient to capture the fluid reality of the survivorship experience [11]. Consequently, healthcare systems often struggle to anticipate and address patients’ evolving needs, leading to gaps in care and unmanaged suffering.

This gap highlights a critical bottleneck in current symptom management paradigms. While the importance of symptom control is widely acknowledged, the clinical approach often remains reactive and episodic rather than proactive and continuous [12]. The transformation of the medical model towards a biopsychosocial perspective necessitates a deeper, more nuanced understanding of the patient’s lived experience to develop effective, personalized interventions [9, 10]. Prior qualitative work, including our own cross-sectional study, has established foundational themes in symptom perception and management dilemmas [11]. However, a pivotal aspect remains underexplored: the longitudinal trajectory of how survivors themselves recognize, interpret, and cope with their symptoms over time and how their needs for support correspondingly change.

To address this knowledge gap, this longitudinal phenomenological study aims to conduct an in-depth exploration of the symptom trajectories in glioma survivorship. By tracking the lived experiences of survivors over time, this research seeks to move beyond a static catalog of symptoms to illuminate the dynamic processes of symptom recognition and interpretation, the diversity in long-term coping strategies, the clinical dilemmas in symptom modulation, and the resultant need for a comprehensive symptom intervention framework. The findings are expected to provide a robust theoretical foundation for developing dynamic, personalized nursing interventions that can better meet the unmet needs of glioma survivors, ultimately aiming to reduce their symptom burden and enhance their quality of life.

## Methods

This study employed a descriptive phenomenological approach, grounded in the philosophical traditions of Husserl, to explore the lived experiences of glioma survivors. A longitudinal, qualitative design with semi-structured interviews was adopted to capture the essence and evolution of symptom trajectories over time.

### Study setting and participant selection

Participants were recruited via purposive sampling. The recruitment period lasted from November 2024 to November 2025. Recruitment was carried out in a large tertiary Grade A specialized cerebrovascular hospital in Central China. Serving as a regional medical center, this hospital covers an extensive population and admits a large number of neuro-oncology patients. Therefore, the enrolled participants can well represent the population of glioma survivors in this region.

### Inclusion and exclusion criteria

Potential participants who met the inclusion and exclusion criteria were screened by neurosurgeons. Eligible participants were adult patients (≥ 18 years) who met the following criteria:

- Inclusion criteria: (1) Diagnosed with primary glioma (WHO grades I–IV), (2) at any disease phase (e.g., newly diagnosed, stable, progressive, or recurrent disease) and having received or currently receiving relevant treatments (e.g., surgery, radiotherapy, chemotherapy), (3) self-reported experience of at least one glioma-related symptom (e.g., headache, seizure, cognitive deficit, motor dysfunction, fatigue, emotional distress) within the past 3 months, (4) proficient in Mandarin Chinese for effective communication, (5) voluntarily provided written informed consent
- Exclusion criteria: (1) Presence of severe cognitive impairment or psychiatric conditions that could compromise interview reliability, (2) at the end-of-life stage with an estimated survival of less than 3 months or in a physical state too frail to tolerate the interview, (3) concurrent participation in other interventional studies that might influence symptom management, (4) unwillingness to have the interview audio-recorded

The study began sampling at baseline (T1) and continued until data saturation was reached—that is, no new themes or subthemes emerged from the data. Preliminary data saturation was achieved upon inclusion of the 11th participant, with one additional participant interviewed to further verify data redundancy and the absence of new information, ensuring sufficient sampling. In this study, sample dropout was defined as failure to complete subsequent interviews due to refusal, severe impact on daily life, or death.

### Data collection

This study employed a semi-structured interview method for longitudinal data collection. Based on the characteristics of the disease, three time points were set after clinical observation, literature review, and expert consultation: initial diagnosis (T1), 1 month post-surgery (T2), 3 months post-surgery (T3). The interview outline was jointly developed by the research team, whose members possess both extensive clinical practical experience and solid expertise in qualitative research. The team includes neurosurgical specialist nurses, master’s students in nursing, and doctoral candidates in nursing. All members have more than 5 years of experience in the field of neuro-oncology and have received systematic and standardized training in qualitative research methods. Prior to formal data collection, two glioma patients were selected for pilot interviews, and the interview outline was revised and optimized based on the feedback. The complete interview outline is detailed in Table 1.

**Table 1:** Guiding questions for in-depth interviews

1.What symptoms are you currently experiencing? Could you share how these symptoms make you feel?
2.Could you talk about the most profound feelings and experiences you have had over the past week?
3.What are your thoughts on this hospitalization experience?
4.What impacts do you believe the disease has had on you? Could you describe them in detail?
5.What impacts do you think the treatment process has had on you? Could you describe them in detail?
6.What do you do when these reactions or feelings arise?
7.What measures do you think could effectively alleviate your symptoms?
8.What kind of help do you most wish to receive?

The interview location is chosen to be a quiet space free from external disturbances, such as a conference room or an empty hospital ward. The interview time is determined based on the interviewee’s specific schedule (such as outpatient follow-up days or during inpatient chemotherapy) and may be affected by various uncertainties like changes in the patient’s condition or treatment arrangements. Therefore, confirmation is made again the day before the interview. Typically, researchers schedule interviews after the patient’s treatment has concluded and when their emotional state is relatively stable. Each interview lasts between 30 and 60 min. Researchers observe and record nonverbal information, such as the participant’s speech rate, tone, facial expressions, and body movements. In addition, researchers carefully listen to the participant’s statements and employ interview techniques such as summarizing, confirming, and clarifying. After each interview, researchers write a reflective journal.

### Analysis

Within 24 h after each interview, the researcher transcribes the interview recording into a Word document. Another researcher then conducts a secondary verification to ensure the accuracy of the content. After confirmation, all the transcribed content is printed and sent back to the interviewee for confirmation. This study uses Colaizzi’s method for data analysis [13]. The specific steps are as follows: First, the researchers repeatedly read the interview records at each time point to gain a comprehensive understanding of the material; then, based on this, they screen and extract all meaningful statements directly related to the patients’ symptom experiences, interpret and condense them, and distill the underlying units of meaning behind the statements; next, similar content is categorized to gradually form themes; finally, an overall description is made focusing on the phenomenon of “how symptoms change over time,” extracting core conclusions and presenting the complete symptom experience of patients from diagnosis to returning to daily life.

To ensure the rigor of the research process and the credibility of the research results, this study employed multiple triangulation strategies for quality control [14]: data coding and analysis were independently conducted by two researchers, and regular project team meetings were held for discussion and reflection. Team members collectively compared different interpretative approaches, examined inherent subjective assumptions, and continuously supplemented, revised, and refined the thematic content, thereby minimizing analytical bias to the greatest extent and enhancing the explanatory power of the research conclusions.

### Ethics approval and consent to participate

The authors respected the Helsinki Declaration [15]. This study was approved by the Institutional Review Board of Henan Provincial People’s Hospital (Approval No. HUSOM2022-382). It forms part of the larger research project, “Dynamic Study of Glioma Patients’ Symptom Group and Construction of a Profile-Based Symptom Management Nursing Intervention Program.” All participants provided written informed consent after a thorough explanation of the study procedures.

## Results

### Participants

Twelve participants were recruited for the study. To protect the privacy of the interviewed subjects, the 12 selected surviving glioma patients were numbered from P1 to P12. Participant characteristics are provided in Table 2: their median age was 51 years (range 31–69), and 58.4% were men. This is an educated group, of which 41.6% (*n* = 5) completed 4-year college. All participants are employed and married with children.

**Table 2:** Characteristics of surviving glioma patients

No.	Age (years)	Gender	Education level	Occupation	Illness duration	Grade (G)	tumor recurrence	Treatment modality	Average interview duration
P1	32	M	University	Worker	3 years	2	Y	**b**	35.12
P2	57	M	Junior college	Self-employed	1 month	1	**N**	**a**	36.59
P3	69	M	University	Teacher	1 week	4	**N**	**c**	49.19
P4	36	M	University	Self-employed	2 months	2	**N**	**a**	41.34
P5	52	F	Junior high school	Self-employed	5 years	1	Y	**a**	37.46
P6	68	M	High school	Self-employed	2 weeks	3	**N**	**b**	36.29
P7	60	M	University	Teacher	1 year	2	**N**	**b**	41.20
P8	41	M	University	staff	1 months	2	**N**	**a**	30.17
P9	66	F	Junior high school	Farmer	2 years	1	Y	**b**	32.48
P10	49	F	Primary school	Self-employed	1 year	2	**N**	**a**	38.56
P11	31	F	Junior high school	Business operator	1 months	3	**N**	**b**	35.07
P12	52	F	Primary school	Civil servant	5 years	1	Y	**b**	31.14

*F* female, *M* male. *Y* yes, *N* no. a, surgery; b, surgery + chemotherapy; c, surgery + radiotherapy and chemotherapy

### Themes

The analysis revealed four core themes characterizing the symptom trajectory of glioma survivors (Table 3): (1) self-perception and interpretation of the disease, (2) differentiated coping with symptoms, (3) practical dilemmas in symptom management, (4) core needs in symptom management.

**Table 3:** Themes and subthemes

Themes	Sub-themes	
Self-perception and interpretation of the disease	Initial symptom experience	
	Initial experience of diagnosis and treatment	
Differentiated coping with symptoms	Adopt positive coping	
	Engage in negative coping	
Practical dilemmas in symptom management	Lack of knowledge about the disease	
	Encounter difficulties in diagnosis and treatment	
	Heavy economic burden	
	Work limitations	
Core needs in symptom management	Health-related information needs	
	Diverse support needs	Emotional support needs from family and peers
		Care and support needs from healthcare professionals
		Social resource support needs

### Theme 1: Self-perception and interpretation of the disease

#### Sub-theme 1: Initial symptom experience

Symptoms induced by gliomas are primarily determined by the tumor’s size, location, and growth rate, often presenting as a range of physical discomforts. These symptoms not only cause significant distress to patients but also impair their daily functioning. Common symptoms reported by participants in the study include headache, fatigue, drowsiness, nausea, vomiting, and decreased physical capacity. However, due to individual variations in tumor characteristics, the specific manifestations of symptoms are heterogeneous.

P6: My head keeps aching on and on and nothing seems to ease it. I also constantly feel nauseous with the urge to vomit and cannot bring myself to eat anything. (68 years old, male, T1)

P1: I can barely eat anything! Every time I try to eat, I feel super nauseous, and I’ve still got this headache that won’t go away. (32 years old, male, T3)

P2: I’m feeling dizzy, and half my face is numb. Before I came to the hospital, I was so tired I could barely move—plus I had really bad nausea and was throwing up nonstop. (57 years old, male, T1)

P7: My left hand and foot are all swollen. Walking feels like stepping on cotton—totally wobbly and hard to get around. I can barely move them properly. Getting up, dressing, washing and other daily tasks are quite arduous. I fear falling when walking alone. I am unable to do most housework and generally rely on my family members to assist me with daily care. (60 years old, male, T3)

#### Sub-theme 2: Initial experience of diagnosis and treatment

During hospitalization, patients often face multiple physiological challenges: not only do they experience significant decline in physical function and a relatively high incidence of chemotherapy-related side effects, but various symptoms also tend to present as overlapping syndromes. Meanwhile, during routine nursing procedures including drainage tube removal, dressing change, wound suture, and nasogastric tube insertion, some patients report discomfort such as headache and wound pain.

P5: It was particularly painful when the tube was removed, and the subsequent suture was also agonizing. (52 years old, female, T2)

P4: I had a headache when the drainage tube was removed and during dressing changes. (36 years old, male, T2)

P2: “Although it’s uncomfortable, there are no alternative treatment options, and I can’t eat anything anyway. It’s just a routine procedure, and I can tolerate it.” (57-year-old male, T3)

The patient suffered from impaired eating function caused by primary intracranial lesions, such that nutritional intake via oral feeding failed to meet his daily physiological requirements. Clinically, nasogastric intubation was performed to deliver enteral nutrition support as well as therapeutic medications through the tube. Despite the physical discomfort induced by intubation, the patient fully recognized the clinical necessity of this intervention and chose to endure the discomfort and cooperate with treatment.

Notably, despite experiencing varying degrees of wound pain or procedure-related discomfort, most patients actively cooperated with nursing care, ensuring the smooth progression of all diagnostic and therapeutic procedures.

### Theme 2: Differentiated coping with symptoms

For glioma patients, regardless of their disease stage or the treatment modalities they undergo—such as surgery, radiotherapy, or chemotherapy—they inevitably confront numerous physical and psychological challenges. When it comes to disease management, particularly in the context of accurate diagnosis and standardized treatment, many patients exhibit distinct attitudes shaped by factors including their sociocultural backgrounds and psychological states.

#### Sub-theme 1: Adopt positive coping

When symptoms manifest, some patients first adopt a positive mindset to accept the illness, perceiving it as understandable and controllable. They then proactively seek effective and reliable resources to conduct symptom management and alleviate physical discomfort.

P3: The hospital where I am currently staying boasts a high standard of medical care and state-of-the-art equipment. I have complete confidence in the doctors’expertise, the advanced technology, and the quality of the medical services provided. I am certain that by cooperating fully with the medical team, my condition will improve, and I will recover as soon as possible. (69 years old, male, T3)

In addition, an optimistic coping mindset not only influences the individual but also inspires other patients in the same ward. As a 60-year-old male patient stated:

P7: “In our ward, the youngest patient is only 6 years old, and the oldest is over 80. We all have the same illness, yet they are all particularly resilient, optimistic, and positive. Their attitude towards the disease has deeply inspired me. (60-year-old male, T3)

#### Sub-theme 2: Engage in negative coping

Some patients may still be trapped in negative emotions due to various factors. The shift in family focus and increased burden leads to a negative state such that symptoms last longer or worsen with more severe physical and psychological consequences.

P5: “Since I fell ill, all family affairs have been put aside. Our business had to be suspended and there is no one to take care of the children. Everything in the family has to center around me. I feel that I alone have become a burden to the whole family,” she sighed. (52 years old, female, T3)

Furthermore, glioma patients often fall into a state of helplessness after diagnosis, with social avoidance behaviors and psychological distress being particularly prevalent. This not only directly triggers various psychological and emotional problems but also increases the risk of mental health conditions such as anxiety and depression. Consequently, patients may adopt a negative attitude toward disease management, which in turn adversely affects the treatment process and rehabilitation outcomes.

P12: Sometimes this illness hits with sudden convulsions—I’d never dare go out alone, scared to death there might be some kind of accident! (52 years old, female, T3)

P11: No doctor can give a definite answer as to whether the tumor will recur or progress later. Every night when I lie in bed, my mind keeps wandering uncontrollably. I am constantly terrified that something terrible might happen unexpectedly; I live in constant anxiety and can never feel at peace. (31 years old, female, T3)

P9: I am reluctant to go out and meet relatives and friends, because I worry they will learn about my brain tumor and talk about me behind my back. So I choose to stay at home most of the time, and gradually I have no desire to interact with other people anymore. (66 years old, female, T3)

### Theme 3: Practical dilemmas in symptom management

#### Sub-theme 1: Lack of knowledge about the disease

Disease-specific knowledge serves as the foundation for the early identification and monitoring of symptoms, as well as the implementation of symptomatic treatment. However, the majority of patients possess limited understanding regarding the prevention and management of glioma and the effective approaches for the early detection of oncological diseases. Furthermore, their access to such information is predominantly via the Internet, resulting in a lack of systematic and personalized disease-related knowledge.

P3: My right side of the head hurts a bit. The doctor came over and gave me an injection, but I’m worried—will this shot affect my upcoming surgery? Besides, I was taking aspirin enteric-coated tablets before being admitted to the hospital. Could this medicine also interfere with the surgery? (69 years old, male, T1)

P4: I’m so scared this illness will come back a second time! I saw a lot of people saying online that if it relapses again, things will get really serious. Brain surgery can also have an impact on the brain, and I’ve been feeling uneasy about it nonstop. (36 years old, male, T3)

#### Sub-theme 2: Encounter difficulties in diagnosis and treatment

Glioma patients often encounter difficulties in diagnosis and treatment due to the complexity of the disease.

P9: It’s been days now, and we still haven’t gotten a regular hospital bed! All the other patients next to us moved to regular beds ages ago, but we’re still stuck on this extra bed outside, making do. Right now, we don’t want anything else—we just hope to get the surgery done as soon as possible! (66 years old, female, T1)

P2: I first went to the hospital ’cause I felt off, but every hospital gave me different test results. None of them could give me a definite diagnosis, and to this day, I still don’t know what’s really wrong with me! (57 years old, male, T1)

#### Sub-theme 3: Heavy economic burden

The costs associated with oncology treatment, such as diagnostic tests, surgery, medications, adjuvant therapy, and review, can be financially burdensome to patients. The long treatment duration and frequent hospital visits can cause patients to stop working and lose their source of income. Patients’ out-of-pocket costs vary widely because of the significant variation in charges at different levels of hospitals, as well as the different types of health insurance coverage.

P4: I’ve got three kids to feed at home, tons of daily expenses piling up, plus two elderly family members to take care of. Life’s pressure is huge—I’m desperate to earn money to support the family! (36 years old, male, T3)

P1: I got married ages ago! Both my sons are still little, and I’ve got a 30-year-old brother who’s not married yet. My grandparents are already in their 70 s or 80 s too. Just for this illness, I’ve already spent over 100,000 yuan in Beijing, and I estimate I’ll have to shell out another 70,000 to 80,000 yuan next! (32 years old, male, T3)

#### Sub-theme 4: Work limitations

Adverse reactions induced by the disease, the risk of tumor recurrence, and the need for medical care lead to significant work limitations among patients. Specific manifestations include inability to attend work normally, work interruption due to seeking medical treatment across regions, and occupational access barriers caused by employment discrimination. These consequences further result in loss of income and impairment of social functioning.

P1: I can’t go anywhere now, and I can’t even go out to work. Whenever I feel unwell, I have to stay in the hospital lying down! (32 years old, male, T3)

P8: If I hadn’t gotten sick, I definitely could’ve found a good job! But bad luck—this illness hit me, and no one dares to hire me. Companies are scared that if I suddenly feel dizzy or have an episode, I’ll get them into trouble! (41 years old, male, T3)

### Theme 4: Core needs in symptom management

#### Sub-theme 1: Health-related information needs

The subtheme of health-related information needs primarily encompasses patients’ demands for personalized treatment plans, follow-up protocols, and rehabilitation guidance. When confronted with an unfamiliar disease, patients exhibit a heightened thirst for knowledge and a desire for specific, actionable information about their condition.

P4: Will we receive follow-up care after discharge? What is the specific follow-up process? (36 years old, male, T2)

P5: I hope doctors can provide detailed information on the available treatment options and the expected treatment outcomes. (52 years old, female, T1)

P11: I wish doctors and nurses would share more disease-specific health guidance. I want to know how to care for myself at home and what precautions I should take. (31 years old, female, T2)

#### Sub-theme 2: Diverse support needs

**2.1 Emotional support needs from family and peers**

Glioma treatment is characterized by high complexity, and most patients tend to fall into a state of confusion and helplessness after diagnosis. Family companionship, as a core psychological pillar, provides patients with crucial emotional comfort and psychological counseling. Meanwhile, the care and understanding from friends serve as an important supplement to patients’ psychological support system, effectively alleviating the sense of loneliness caused by the disease. Most interviewees explicitly expressed an urgent need for companionship from family and friends, gaining psychological strength to cope with the disease through in-depth communication and emotional resonance.

P8: Chatting with friends eases my anxiety a lot—I feel totally at ease venting to them and spilling my guts. We’ve agreed to meet up again once I’m fully recovered. Hope this research reaches more people going through similar stuff, so we can help each other out and have each other’s backs! (41 years old, male, T3)

P9: When my family sits down to chat with me earnestly, I feel particularly at peace and comfortable. (66 years old, female, T3)

**2.2 Care and support needs from healthcare professionals**

During hospitalization, the mutual trust established between healthcare professionals and patients through professional interactions can be transformed into strong psychological support for patients to cope with disease symptoms. The targeted guidance and humanistic care provided by healthcare professionals based on their expertise not only effectively alleviate patients’ physical pain but also reduce their psychological burden, helping patients face the disease with a more positive mindset.

P11: When my headache hits and it’s totally unbearable, the doctors and nurses always patiently show me what to do. They even chat with me to help me feel better, and the pain isn’t nearly as bad afterward~ (31 years old, female, T3)

**2.3 Social resource support needs**

Social support refers to a psychological experience of mutual recognition and satisfaction among individuals formed through social interaction and mutual influence, which plays a vital role in regulating the physical and psychological status of glioma patients. Due to the heavy economic pressure and life burden brought by disease treatment, interviewees generally hope to gain social attention and resource support, with economic support being the core demand to alleviate the financial strain caused by medical expenses. Meanwhile, they anticipate the formation of a support system featuring group mutual assistance and social support.

P8: Ordinary families spend a fortune on medical care—it’s like money flowing away nonstop! Hope hospitals can offer some help, and what we need most urgently is financial support. May us fellow patients help each other out, and also get more care and support from all sides of society!? (41 years old, male, T3)

## Discussion

This study focused on hospitalized glioma survivors, aiming to establish, describe, and analyze their symptom management experiences and needs. As the first domestic research to concentrate on the psychological and symptomatic experiences of this population, it fills the gap in relevant studies. The results show that glioma patients have multi-dimensional symptomatic experiences: physically, they suffer from limited physical activity and treatment side effects; psychologically, they experience negative emotions such as anxiety and fear of recurrence; socially, changes take place in their family roles and social support status. Their needs for symptom management span multiple life dimensions: individual, existential, family, cultural, community, and national. Glioma exerts comprehensive and persistent impacts on patients themselves, their families, and support systems, forming a complex web of phenomena. Following diagnosis, patients’ life trajectories are profoundly altered, requiring them to learn to adapt to and manage physical pain, fatigue, and emotional distress, ultimately reconstructing a “normalized life.” Additionally, some patients resist being pitied by others. Based on these insights, oncology nursing must adhere to the core principle of sensitively perceiving, listening to, and appropriately responding to patients’ needs, while constructing a holistic and multi-faceted care model. Since other specialists only provide phased targeted treatment, clinical nurses are suitable to lead the construction of a psychosocial oncology care system tailored to China’s practical conditions, so as to effectively meet the diverse care needs of glioma survivors. Benefiting from uninterrupted care throughout the entire disease course, nurses can keep abreast of patients’ dynamic physical, psychological, and social needs in real time; coordinate multiple medical teams; integrate various specialized services into systematic individualized care; and carry out regular needs assessment and long-term follow-up management continuously.

With the shift toward the biopsychosocial medical paradigm, symptom management has emerged as a core topic in clinical research. However, effective resources for managing symptoms in glioma patients remain scarce. Glioma patients typically bear a significant symptom burden, with clinical manifestations including common physical symptoms such as headache, dizziness, nausea, vomiting, tinnitus, and papilledema. In severe cases, they may also experience epilepsy and various physical dysfunctions. Taking tinnitus as an example, persistent tinnitus interferes with patients’ daily verbal communication and tends to trigger social withdrawal and self-isolation over time. On the one hand, it disrupts patients’ established stable social relationships; on the other hand, it prevents them from forming new social bonds, which ultimately imposes severe secondary psychological pressure on patients. Additionally, symptoms like fatigue, depression, and anxiety can persist throughout the entire disease trajectory. Notably, some symptoms form a vicious cycle (e.g., fatigue leads to reduced physical activity, which further exacerbates anorexia and physical deterioration, ultimately worsening fatigue). Moreover, frequent nausea and vomiting can aggravate the patient’s condition and reduce the overall efficacy of treatment [16, 17]. In addition, compression of brain tissue caused by intracranial space-occupying lesions may lead to cognitive dysfunction and personality changes in some patients, including memory impairment, poor concentration, and disorientation, which directly impair their ability to work and perform activities of daily living. Certain patients also develop personality alterations: some become irritable and prone to emotional outbursts, whereas others turn withdrawn, apathetic, and slow in response.

Based on these observations, healthcare professionals, particularly nurses, must prioritize symptom management for glioma patients. This not only serves as a key measure to alleviate patient discomfort and address their symptom-related needs but also constitutes a critical prerequisite for patient-centered holistic care for glioma patients [18]. Nurses play a central role in symptom management: through high-quality symptom assessment and targeted interventions, they can not only effectively reduce patients’ symptom burden and improve their quality of life but also help patients and their families establish a positive treatment mindset, thereby providing support for optimizing the diagnosis and treatment outcomes of glioma patients [19].

The findings of this study indicate that compared with patients with other types of tumors, glioma patients exhibit more pronounced disease-specific characteristics—apart from being prone to cognitive impairment and various disease-related complications, they also commonly face issues such as poor treatment response and suboptimal prognosis [20]. The interplay between these disease features and multiple contributing factors exposes patients to severe psychological distress: from the diagnostic phase when they become aware of the life-threatening nature of the disease, the unfavorable prognosis and uncertain disease progression trigger feelings of fear and severe anxiety in patients. Meanwhile, insufficient disease-related knowledge among patients and the social stigma associated with the illness further amplify their negative psychological tendencies [21], thereby fostering adverse emotions such as self-doubt, low mood, and irritability. These psychological problems not only increase the risk of developing mental health disorders such as depression but also impair patients’ normal social functioning, rendering them unable to proactively and effectively engage in disease self-management [22, 23]. Additionally, the persistent stress arising from disease diagnosis and treatment further exacerbates patients’ negative emotions and depressed mindset, ultimately exerting an adverse impact on their health outcomes.

Relevant research has confirmed that when patients actively mobilize their subjective initiative and adopt positive coping strategies in response to the disease, they can effectively alleviate physical and psychological discomfort and improve their quality of life [24]. Based on this, nurses should integrate psychological interventions into the holistic care plan for glioma patients: by dynamically tracking and assessing the trajectory of patients’ psychological changes [25], applying professional psychological theories and methods to targetedly guide patients’ mental activities, and implementing individualized psychological intervention measures, nurses can help patients develop self-regulation capabilities and maintain a positive and optimistic emotional state. This intervention approach not only effectively reduces the severity of patients’ symptom burden but also alleviates the physical pain and psychological distress caused by the disease [26], providing solid support for optimizing the prognostic outcomes of glioma patients.

Our study indicates that glioma patients face four major practical challenges in symptom management: lack of disease knowledge, heavy financial burden, work limitations, and difficulties in diagnosis and treatment. These challenges are not isolated but intertwine and influence each other in clinical settings, forming a vicious cycle of “insufficient cognition → limited treatment → economic loss → social isolation.” This predicament severely impacts patients’ symptom control outcomes and quality of life [27, 28].

This finding provides clear practical guidance for clinical care and social support: clinical nursing must move beyond traditional symptomatic approaches (“treating the head for a headache, the foot for a footache”) and instead build a multidimensional, collaborative intervention model grounded in patients’ real-life contexts. This model should be “problem-oriented + needs-matched.” Cognitively, standardized public education must be replaced with tailored approaches. Considering patients’ varying ages and educational backgrounds, information should be delivered through accessible methods like bedside one-on-one guidance, short educational videos, and peer experience sharing sessions. This transforms disease knowledge, medication precautions, and symptom monitoring methods into practical, patient-understandable content that genuinely enhances self-management capabilities [29]. In medical services, address pain points in the diagnostic process by optimizing bed reservation systems, establishing multi-hospital information-sharing platforms, and promoting standardized MDT protocols for glioma treatment to reduce patient travel between hospitals and diagnostic delays.

Through interviews, this study found that glioma patients have two core needs in symptom management: health-related information needs and diverse support needs. These needs directly reflect the practical dilemmas and psychological expectations of patients when coping with the disease. Health-related information needs are the fundamental demands of patients facing an unfamiliar illness, focusing on three key dimensions: personalized treatment plans, follow-up protocols, and home-based rehabilitation guidance. This suggests that information support for cancer patients should be provided throughout the entire treatment cycle.

Furthermore, the study revealed that the diverse support needs of glioma patients exhibit multi-level and comprehensive characteristics, encompassing emotional support from family and friends, care and support from healthcare professionals, and social resource support [30]. This finding indicates that healthcare providers need to deliver continuous supportive services [31]. The transition period from hospital discharge to returning home is an extremely sensitive stage for glioma patients and their families [32]. The interviewed patients had already experienced anxiety regarding subsequent treatment and post-discharge rehabilitation management during hospitalization, expressing a strong desire for specialized personnel to regularly follow up and assess their rehabilitation progress. Even after patients are discharged, continuous nursing services should be promptly provided [33]. Healthcare teams should track the occurrence of symptoms post-discharge through telephone calls, text messages, or home visits, offer professional advice and management strategies, provide psychological support when necessary, and encourage family members and friends to participate in care and offer ongoing emotional companionship. In this way, patients can receive comprehensive and continuous physical and psychological care.

## Limitations

The main potential limitation of this qualitative study includes possible confirmation bias or possible missing data. The small sample size of this study, in which all participants were recruited from a tertiary hospital in Henan Province, China, is not generalizable, and what they experienced may not be representative of those living in other parts of the world or receiving oncology treatment at other hospitals. Although all participants were insured and received oncology treatment, most had low incomes and education levels and may not be representative of glioma patients with higher incomes and education levels.

Despite appealing these factors, our study provides evidence that illuminates the impact of glioma on Chinese patients, as well as on their families and others and makes a significant contribution to the qualitative literature on Chinese glioma patients. This helps clinical nurses understand the real-life experiences of hospital survivors of gliomas, their perceptions of related diseases, and their nursing needs. In the future, there is a need to focus on the symptom management needs of glioma patients during hospitalization so that nurses can provide appropriate, targeted, and comprehensive quality care and precision nursing services to patients promptly to provide a basis for the formation of a system of care for glioma patients.

## Conclusion

In this study, semi-structured interviews were used to deeply explore the nursing needs of symptom experience and symptom management of living glioma.

## Data Availability

Due to the principle of data confidentiality, the datasets generated and analyzed in this study are not publicly available. Upon reasonable request, the datasets can be obtained from the corresponding author.
